# P144 a Transforming Growth Factor Beta Inhibitor Peptide, Generates Antifibrogenic Effects in a Radiotherapy Induced Fibrosis Model

**DOI:** 10.3390/curroncol29040217

**Published:** 2022-04-12

**Authors:** Sebastián Cruz-Morande, Javier Dotor, Mikel San-Julian

**Affiliations:** 1 Orthopaedic Surgery and Traumatology Department, Clínica San Miguel, Beloso Alto 36, 31006 Pamplona, Spain; 2 DISIT Biotech, Fuenlabrada Hospital, 28942 Madrid, Spain; jdotor@disitbiotech.com; 3 Orthopaedic Surgery and Traumatology Department, Clínica Universidad de Navarra, Av. Pio XII 36, 31008 Pamplona, Spain; msjulian@unav.es

**Keywords:** soft tissue sarcomas, radio-induced fibrosis, brachytherapy, transforming growth factor-beta1 (TGF-β1), Disitertide, Smad 2/3

## Abstract

Radiation-induced fibrosis (RIF) is a severe side effect related with soft tissues sarcomas (STS) radiotherapy. RIF is a multicellular process initiated primarily by TGF-β1 that is increased in irradiated tissue, whose signaling leads to intracellular Smad2/3 phosphorylation and further induction of profibrotic target genes. P144 (Disetertide©) is a peptide inhibitor of TGF-β1 and is proposed as a candidate compound for reducing RIF associated wound healing problems and muscle fibrosis in STS. Methods: A treatment and control group of WNZ rabbits were employed to implement a brachytherapy animal model, through catheter implantation at the lower limb. Two days after implantation, animals received 20 Gy isodosis, intended to induce a high RIF grade. The treatment group received intravenous P144 administration following a brachytherapy session, repeated at 24–72 h post-radiation, while the control group received placebo. Four weeks later, affected muscular tissues underwent histological processing for collagen quantification and P-Smad2/3 immunohistochemistry through image analysis. Results: High isodosis Brachytherapy produced remarkable fibrosis in this experimental model. Results showed retained macro and microscopical morphology of muscle in the P144 treated group, with reduced extracellular matrix fibrosis, with a lower area of collagen deposition measured through Masson’s trichrome staining. Intravenous P144 also induced a significant reduction in Smad2/3 phosphorylation levels compared with the placebo group. Conclusions: P144 administration clearly reduces RIF and opens a new potential co-treatment approach to reduce complications in soft tissue sarcoma (STS) radiotherapy. Further studies are required to establish whether the dosage and timing optimization of P144 administration, in different RIF phases, might entirely avoid fibrosis associated with STS brachytherapy.

## 1. Introduction

Soft tissue sarcomas (STSs) are uncommon tumors of mesenchymal origin, with different subtypes having a different prognostic profile. STSs most commonly arise in the extremities, but can also occur in the trunk and retroperitoneum [1].

STSs account for 1% of all adult malignancies, with a global incidence of 180,000 cases per year and a mortality of 80,000 patients/year [2]. Extremity soft tissue sarcomas (ESTSs) are diagnosed frequently with a delay, due to the painless presentation and the rarity of the disease. For this reason, they often produce large tumor masses, with a mean tumor diameter of 10 cm at time of diagnosis [3].

Treatment includes surgical resection in combination with radiotherapy. Limb-preserving surgery combined with radiotherapy has dramatically improved the local control of soft tissue sarcoma patients [2,4,5]. However, it still carries a substantial risk of acute side effects, such as fatigue, nausea, vomitous, diarrhea, hair loss, or skin or mouth damage, and long-term side-effects that depend on the irradiated tissue and might include heart complications, breast size changes, damage in the lungs, brachial plexopathy, fertility problems together with changes in sexual life, and cystitis [6]. Post-radiotherapy fibrosis in the treatment of childhood soft tissue sarcomas occurs in 80% of patients, in different degrees of involvement [7], and 95% of patients have radiodermatitis, which has a similar pathophysiology [8].

Brachytherapy is a modality of radiotherapy used in the treatment of the soft tissue sarcomas [9,10]. This treatment frequently induces fibrotic processes in the tumor surrounding tissues like skin, the skeletal muscle and fascias [11,12], similar to other radiotherapy modalities. The administration of an early radiation within the first postoperative month is associated with the highest morbidity, whereas complication rates decrease with time. On the other hand, postponed radiation may lead to oncological compromises [13].

Although radio-induced fibrosis (RIF) closely resembles the chronic healing of a traumatic wound, it is subject to irradiation related disturbances, because all the cells and extracellular components of the irradiated volume tissues have been affected.

Fibrosis is essentially involved in the genesis of late reactions in slowly renewed healthy connective tissue with a non-compartmentalized structure, such as the dermis and subcutaneous tissues [14], or vasculo-connective parenchymal tissue [15].

Fibrosis is characterized by the activation and increase of an excessive number of activated fibroblasts, resulting in the deposition of extracellular matrix proteins such as collagen and impairment of normal tissue architecture. Although fibrosis is a physiological part of wound-healing processes, the excessive accumulation of collagen and other extracellular matrix components can lead to the destruction of normal tissue architecture and loss of function [11]. Recent studies have reported that cells other than fibroblasts also contribute very significantly to the appearance of fibrosis. Among these cells we find the macrophages resident in the connective tissue, which, as is well known, play a very important role in maintaining and amplifying the inflammatory response. This important role is due at least in part to the fact that macrophages are an important source of TGF-β and, in turn, this TGF-β contributes to the increased production of reactive oxygen species, which is closely linked to increased inflammation and the appearance of fibrosis [16,17].

Abnormal fibroblast proliferation and differentiation is considered central to fibrosis. RIF is a multicellular process that begins with the induction of and interaction between multiple growth factors and cytokines [18]. Among these factors, TGF-β1 levels are increased in irradiated mouse skin [19,20] and decrease slowly after irradiation in both pig and human skin [21,22]. 

Following microvascular hard or soft tissue transfer, TGF-β1 is again upregulated in a biphasic manner. The first expression peak on day 3 post operation is due to the enhanced activation of latent TGF-β1 by extracellular enzymes while the second peak of TGF--β1 expression between 14 and 28 days after surgery is the result of de novo synthesis cascade [23]. Its most important signaling receptor TGFBR2 is upregulated in irradiated graft beds as well [24]. TGFβ1 signaling leads to increased nucleoplasmatic shuttling of active Smad2/3 and induction of TGF-β1 target genes in fibrotic healing, which is mainly due to the decrease in cytoplasmatic levels of the inhibitory Smad7. As a consequence, the extracellular matrix is qualitatively and quantitatively altered [24,25]. Some of these alterations are related to Prolyl-hydroxyprolinase-β overexpression that promotes synthesis of collagen I, III, and IV, while the repression of degrading enzymes such as MMP-1 and induction of tissue inhibitors [24,26,27] suppress the degrading pathways. All these molecular events induced by active TGF-β1 generates the deposition of an excessive and dysfunctional extracellular matrix.

TGF-β is a cytokine with a very low half-life, around 2–3 min. It has been demonstrated that upon activation of its receptor, downstream phosphorylation of Smad 2/3 is a good marker of TGF-β pathway activity and it is better than direct measurement of latent/active TGF-β presence [28]. Some drug candidates, such as peptide P144 (Disitertide^®^—TSLDASIIWAMMQN), can inhibit TGβ-1 activity and have been successfully tested in clinical trials for pathological skin fibrosis conditions such as scleroderma [29]

P144 is a poorly soluble hydrophobic peptide derived from the sequence of the extracellular region of TGF-β type III receptor (Betaglycan) and specifically identified to block the interaction of TGF-β with its membrane receptors, blocking TGF-β1 biological activity in different in vitro and in vivo models [3,29,30,31]. P144 inhibits TGFβ1-dependent fibrosis [3] and also has the potential to present enhancing effects over antitumor immunotherapy [31].

In this study, it is proposed that targeting TGF-β1 with the synthetic peptide P144 (DISIT Biotech, Spain) could be an appropriate strategy for reducing the RIF of the muscle and thus reducing wound healing problems, which represent the major cause of complications related with limbs soft tissues sarcomas treatment [32].

## 2. Material and Methods

### 2.1. Animals

This study was approved by the Ethical Committee for Animal Experimentation of our institution (authorization number 032-07) and animal experimentation was conducted in accordance with Spanish and European legislation and approved by the Spanish National Research Council (CSIC).

For the study, adult female and male rabbits (aged 3–4 months, weighing 2.5–3 kg) were used. Rabbits were fed ad libitum with a standard diet and drinking water and controlled following FELASA (Federation of European Laboratory Animal Science Associations) recommendations.

The animals were randomly divided in three groups, namely the experimental model implementation group (*n* = 5), study group treated with P144 (*n* = 6), and placebo group treated with intra venomous (IV) saline vehicle (*n* = 6). Three rabbits were reserved as backup specimens if any complication occurs during the study.

### 2.2. Surgical Technique

The rabbits were intramuscularly anesthetized with a mixture of ketamine (Imalgene^®^ 1000) (35 mg/kg) and Xylacine (Rompun^®^ 2%) (5 mg/kg) before all surgical and irradiation procedures. Injections were administered with a 1 mL syringe and a 25-gauge needle and was repeat if required every 30 min, associated with 0.007 mg Fentanil (Fentanest^®^). After 2–4 min, adopting the aseptic technique, a longitudinal skin incision on the lateral aspect of the left leg was performed. The hamstrings muscle was recognized and a portion of muscle of 2 cm^3^ was resected, then two 6F semiflexible high dose rate brachytherapy catheters were placed as parallel as possible at 1.0 cm in an intramuscular form in the hamstring. Passing in a subcutaneous way to the dorsal aspect of the rabbit thorax, the catheters were secured to the skin by suture stiches and protected with sterilized dressing. As postoperative analgesia, the animals received Ketoprofen (Ketofen^®^ 10 mg/mL), 0.3 mL/kg intramuscularly every 24 h for three days.

### 2.3. Brachytherapy

**Figure 1:**
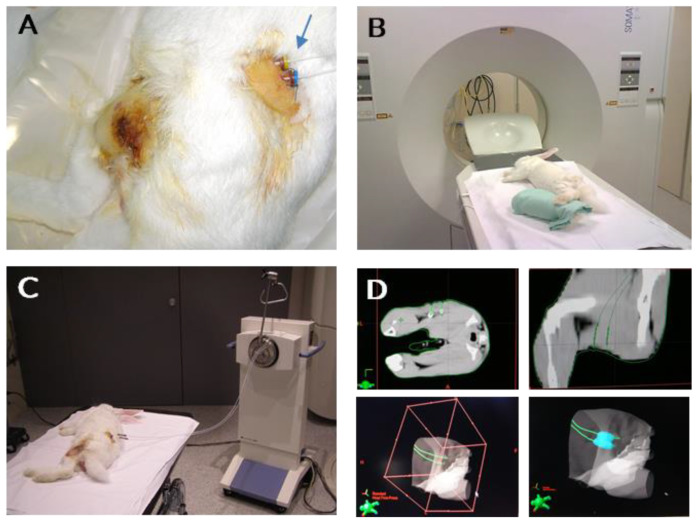
Images of rabbits undergoing brachytherapy procedure. (**A**) Catheters implantation in left posterior rabbit’s limb as brachytherapy source applicator (blue arrow). (**B**) Imaging assurance of correct positioning of catheters through SOMATOM CT Sliding Ganty (**C**) Air pressure control of radioactive seeds delivery and positioning with a GammaMedplus IX (Varian Medical Systems). (**D**) 2D and 3D imaging guided axial visualization for virtual delivery of radioactive sources (BrachyVision™).

After 48 h, a CT-guided brachytherapy planning was performed for each rabbit with the BrachyVision™ Brachytherapy Treatment Planning System (v.8.0, Varian, Palo Alto, CA, USA). Two rabbits of the model development group were irradiated with an Isodosis of 15 Gy, and two with 20 Gy with an Iridium 192 high dose rate (HDR) source in a constant volume of affected tissue (Figure 1). All the rabbits in the study and control group were irradiated with an Isodosis of 20 Gy. Immediately after brachytherapy procedures, the catheters were removed in all the rabbits

### 2.4. Drug Administration

Disitertide^®^ (P144) was manufactured by Polypeptide Group (Strasbourg, France) as the lyophilized peptide was stored at −80 °C before the manipulation peptide vial was tempered to room temperature and then weighed, resuspended in buffer diazonium salt of carbonic acid 0.1 M pH 9.5, and sonicated until a homogeneous solution was obtained. Peptide was IV in the marginal ear veins of the rabbits at doses 10 mg per administration diluted in 10 mL of buffer (approximately 3.5 mg/Kg). As previously mentioned, this dose range was shown to be effective in prior published animal models of inflammation and fibrosis [3,29,33]. Placebo rabbits were injected IV with 10 mL of diazonium salt of carbonic acid 0.1 M pH 9.5.

The first dose was administrated immediately after the radiotherapy and repeated at 24 and 72 h after the first administration.

### 2.5. Sacrifice and Histological Examinations

After 4 weeks, the animals were sacrificed with a lethal dose of barbiturates, followed immediately by a resection of hamstring muscle of the irradiated leg, the muscle samples were embedded in paraffin after an overnight fixation in 4% polyformaldehyde solutions. A series of sections were routinely stained with hematoxylin and eosin, Massons trichrome. The immunohistochemical detection was performed with anti-phosphorylated Smad2/3 polyclonal antibody (Santa Cruz Biotechnology, Santa Cruz, California) adopting a biotin peroxidase-based method (ABC, Vector Laboratories, Burlingame, CA, USA).

### 2.6. Histomorphometric and Immunohistochemical Analysis

Semi-quantitative measurement of the total tissue area, collagen fibers, and positive P-Smad 2/3 cells area was performed using digital images obtained with a Zeiss AxioCamICc3 camera (Plan-Neofluar objective with 0.50 NA) at 20× magnification with an AxioImager.M1 microscope (Zeiss, Oberkochen, Germany).

The quantification was based on collagen fibers stained in blue with Masson’s Trichrome and immunohistochemical staining of P-Smad2/3. AxioVision software was used to conform a mosaic image of the whole muscle tissue sample with of different tissue pictures. We used four sections of each muscle tissue sample. Mosaic images were analyzed using an in-house developed plug-in for Fiji (a distribution of ImageJ) V1.46b. Individual Images were analyzed using an in-house developed plug-in for Fiji (a distribution of ImageJ) V1.48v. Then, images were subjected to threshold to measure the positive staining area of each marker. Mean intensity of staining value was also measured for all threshold areas. P144 effect over P-Smad 2/3 levels in RIF was presented as the positive stained area versus total area ratio in comparison with placebo treated group.

### 2.7. Statistics

The non-parametric Kruskal–Wallis test was used for comparisons between multiple groups and U Mann–Whitney tests were used for comparisons between two groups. A *p*-value of less than 0.05 was considered statistically significant. Statistical analyses of data were performed using GraphPad Prism 9 for Windows (GraphPad Software, San Diego, CA, USA).

## 3. Results

### 3.1. Animal Model

The proposed animal model presents a plausible manipulation and reproducibility. All the procedures were properly tolerated by experimental animals and no surgical related complications were detected. After four weeks placebo and P144 treated groups showed a weight mean increase of 10.4% and 14.1%, respectively, but without being statistically different.

### 3.2. Skin and Articular Range

**Figure 2:**
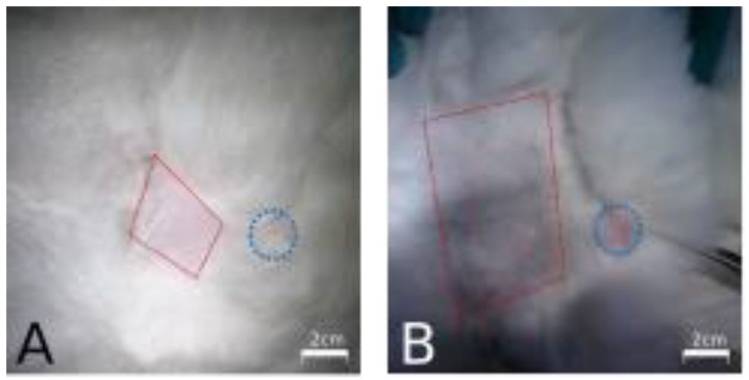
Rabbits skin regions expose to internal brachytherapy. (**A**) Representative picture of a rabbits treated with P144. (**B**) Representative picture of a rabbits treated with placebo. Skin area affected with post brachytherapy alopecia is indicated with discontinued red line square and catheters insertion point is indicated with a discontinued blue circle.

Interestingly, all rabbits receiving P144 present less area and alopecia intensity in the skin region affected with brachytherapy application while the placebo group developed a marked and more extensive alopecia in the same region (Figure 2)

Post operated animals´ legs of all groups underwent a range of motion analysis, and no differences between contralateral legs, hip, and knee were found, discarding surgical affectation of surrounding joints, muscles, and tendons.

### 3.3. Muscle Fibrosis

**Figure 3:**
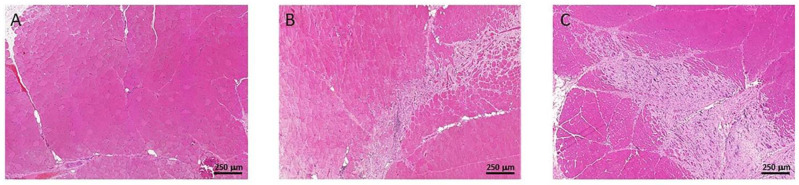
Hematoxylin and eosin staining example 5× showing the difference in muscular organization: (**A**) Normal Tissue, (**B**) P144 group (**C**) Placebo Group.

In both animal groups, different amount of muscle disorganization and loss of the fibrillar patron of the muscle were detected, being qualitatively more evident in the placebo group. The P144 treated group presents more extensive areas with preserved muscle structure in the irradiated tissues associated with less necrosis and a lower presence of collagen deposition with respect to the placebo group (Figure 3).

**Figure 4:**
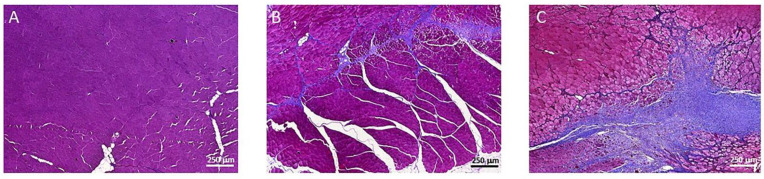
Masson´s Trichrome staining example 10×: (**A**) Normal Tissue, (**B**) P144 group, (**C**) Placebo group.

**Figure 5:**
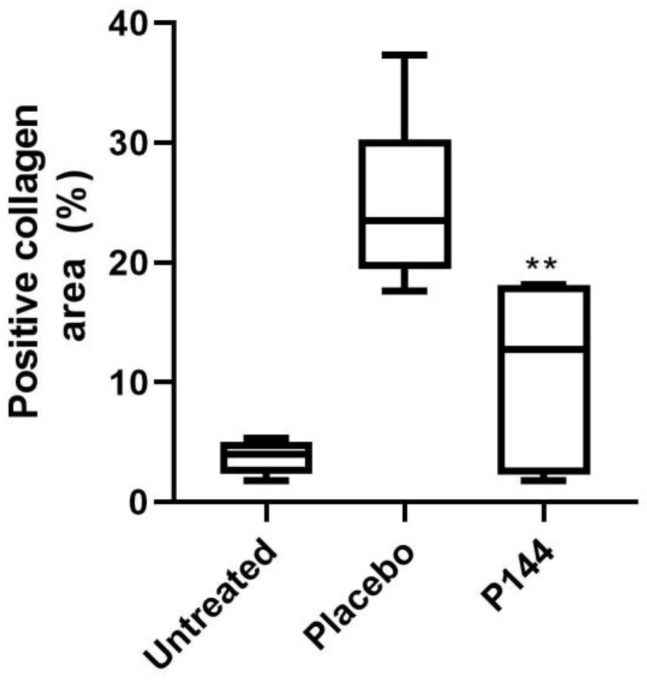
Histological collagen quantification in Masson’s Trichrome stained slides in rabbits muscle tissue that underwent brachytherapy. Untreated, Placebo and P144 treated animals´ collagen tissue content. Statistical significance ** *p* < 0.01 vs. Placebo group.

To evaluate the tissue collagen content in the muscles, a Masson’s trichrome staining was performed, generating a mean collagen area of 11% in the P144 treated group with respect to a 24.9% of collagen-stained areas in muscle of the placebo group (*p* < 0.007) (Figure 4 and Figure 5). The evaluation of collagen area in Masson’s trichrome stained tissue where the brachytherapy catheters were placed showed only 2% of positive area with no differences between groups.

### 3.4. P-Smad2/3 Immunohistochemical Staining

**Figure 6:**
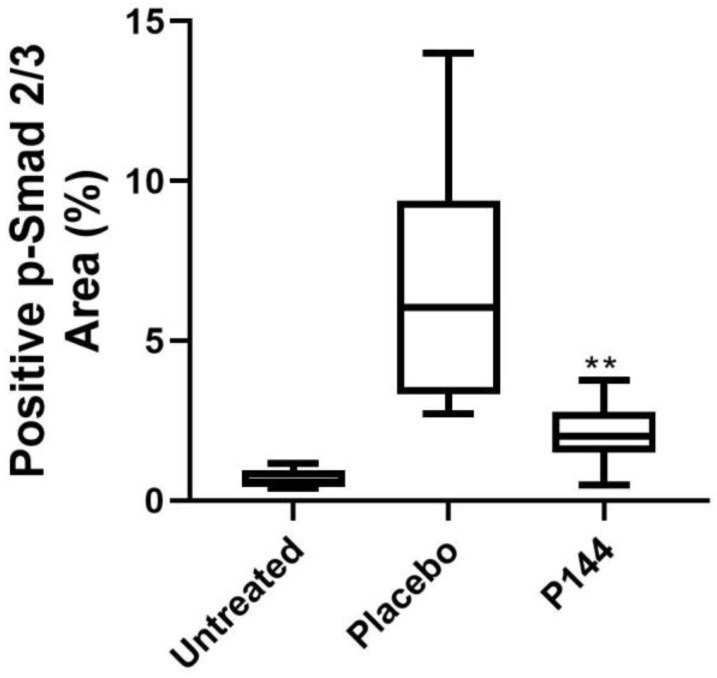
Immunohistochemical positive quantification of P-Smad2/3 in stained slides of rabbits’ muscle tissue that underwent brachytherapy. Placebo vs. P144 treated animals’ TGF-β signaling activation measured as positive detection of p-smad 2/3. Statistical significance ** *p* < 0.01.

**Figure 7:**
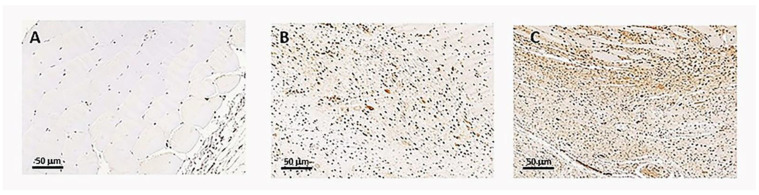
Representative Immunohistochemical positive p-Smad2/3 cells in stained slides of rabbits’ muscle tissue Image 20×. (**A**) Normal Tissue example, (**B**) P144 group example, (**C**). Placebo group.

Intravenous administration of P144 induced a significant reduction in Smad2/3 phosphorylation levels compared to the placebo group (*p* < 0.05) four weeks after brachytherapy as demonstrated in the reduced levels of p-Smad2/3 in the P144 group vs. placebo group (*p* < 0.01) (Figure 6 and Figure 7).

## 4. Discussion

Rodent models are often used to demonstrate the proof-of-principle tracer and therapeutic agent development, but their small size can make radiation dosing and tissue sampling collection challenging. The in vivo model obtained by the resection of muscle fragment and the radiation of the surgical area mimic a tumoral bed in rabbits, resulting in a plausible animal model for the study of the RIF in humans. Different animal models are described in the literature for radiation-induced fibrosis in rodents [34,35] or even in large animals [36], but there is no record in the literature describing an animal model similar to that pointed out in the present work and developed exclusively to evaluate muscular RIF.

In this work, the fibrotic response of limb muscles and surrender tissues to radiotherapy injury were monitored by histological methods, and according to other parameters that represent local and systemic damage cause by radiotherapy.

We found less alopecia in rabbits treated with P144 in the irradiated area, which may be due to the action of the peptide. P144 showed clear antifibrotic activity after topical application in a skin fibrosis mice model [29] and immunohistochemical studies in these P144-treated mice revealed a remarkable suppression of connective tissue growth factor expression, fibroblast SMAD2/3 phosphorylation, and alpha-smooth muscle actin positive myofibroblast development, whereas mast cell and mononuclear cell infiltration was not modified. These data suggested that the topical application of P144, a peptide inhibitor of TGF-β, is a feasible strategy to treat pathological skin scarring and skin fibrotic diseases for which there is no specific therapy. The systemic administration of the same active compound (P144) could exert a relevant antifibrotic effect in skin damage by radiotherapy. Moreover, P144 present anti-inflammatory properties that could protect hair follicles from the initial damage after a brachytherapy session [28].

Radiotherapy causes cellular injury by damaging the DNA and by generating free radicals [11]. Free-radical inactivation of anticoagulatory factors leads to rapid activation of the coagulation cascade following radiation injury. Endothelial cell apoptosis and slow regenerative proliferation result in increased vascular permeability and vessels denuded of endothelium which are prone to thrombosis, intimal proliferation, and eventually obliteration. Physical trauma results in the activation of an acute inflammatory response by stress-sensitive kinases and transcription factors. Pro-inflammatory cytokines, such as tumor necrosis factor-α (TNFα), interleukin (IL)-1, IL-8, and interferon-γ (IFNγ) are synthesized [15]. The termination of the inflammatory response results from the short half-life of these cytokines and anti-inflammatory cytokines, such as transforming growth factor-β (TGFβ), IL-4, IL-10, and IL-13. Inflammation does not resolve adequately following radiation injury because of the overproduction of pro-inflammatory cytokines leading to perturbed intercellular and cell–matrix interactions, uncontrolled matrix accumulation, and fibrosis [37]. This excessive fibrosis is characterized by collagen deposition and microvascular injury of the surrounding tumor healthy tissues, including skin, muscles, soft tissues, and internal organs (lungs, liver, etc.). In this context, collagen fibers represent the major component of the fibrotic extracellular matrix. Excessive collagen synthesis and accumulation was the rationale for pointing to collagen turnover as an activity and severity measure in radiotherapy induced fibrosis.

Collagen deposition is the final marker of the RIF pathophysiological process severely affecting irradiated organs and tissue in a mechanical and functional way. TGF-β/Smad signaling plays an essential role in the pathogenesis of muscular RIF. As P144 is a specific inhibitor of Smad intracellular activation by blocking extracellular TGF-β and inhibiting its interaction with membrane TGF-β receptors, the evaluation of P144 over Smad2/3 phosphorylation in RIF was performed. In this study, the efficacy of an inhibitor peptide of TGF β (P-144), intravenously administered, over irradiated tissue collagen content and TGF-β signaling activation, measured as P-Smad2/3 levels, show a significant lower phosphorylation of SMAD2 in the P144 group. Similar to our results, Disitertide induced significant inhibition of basal pSMAD2 in SNU449 cells [38]. Anscher et al. [35] demonstrated previously that direct interference with the actions of TGF-β can ameliorate the manifestations of the RIF on the lungs by using an anti-TGF-β antibody. Other authors show that black soybean anthocyanins inhibited radiation-induced fibrosis by downregulating TGF-β and Smad3 expression that resulted in a significant reduction in the level of skin injury, epidermal thickness, and collagen deposition after irradiation [39]. These findings are in the same line of our study, in that by the inhibition of the TGF-β, the final results constitute a reduction of collagen deposition in the extracellular matrix. Simultaneously, the direct correlation between P144, TGF-β biological activity inhibition, and fibrosis reduction confirms that intravenous administration of this compound is effective in the prevention for tissue affectation in an animal model representative of human radiotherapy induced fibrosis.

The peptide p144 has been used in different dermal fibrosis models, e.g., for the treatment of hypertrophic scars using a topical form [40] as an alternative route of administration in addition to the intravenous route [29], and also used intravitreally [41]. These results point out that there would be no limits in testing P-144 antifibrotic actions in other organs, such as the lung, where p17, a peptide similar to P144, has been tested showing good results [42]. On the other hand, it is well known that inflammation and fibrosis lead to transdifferentiation of fibroblast in myofibroblast and even favor the transition of epithelial to mesenchyme cells [43,44]. Thus, changes in the cellular profile of the connective tissue after treatment with P144 in RIF models needs to be evaluated in further studies.

These results confirm those obtained in previous research [3,30] regarding the efficacy of a systemic administration of P144 in fibrosis reduction in other kinds of fibrosis models and provide the basis for the clinical interest in a P144 intravenous formulation for further preclinical and clinical development of RIF protective therapy.

In vivo P144 activity against fibrosis is comparable or even superior to other TGF-β inhibitor compounds. In the work of Park et al., the effects of a small molecule inhibitor of TGF-β RI (SKI2162) activity in a model of skin RIF in mice were reported [45]. The effects were partial, and the dosage ranged from 10 to 30 mg/kg, while in the present study P144 is administered in a range of 2–3 mg/kg. Moreover, the intramuscular brachytherapy model is a more severe challenge in damage and tissue response respect skin irradiation. In a similar work, Flechsig et al. showed the effect of other small molecule (LY2109761) inhibitor in a lung RIF murine model, where effects were relevant but also partial and the dosage regimen was 50 mg/kg twice daily for four weeks [46].

There is not direct proof of how p144 may act against sarcomas yet. However, several studies in other type of cancers point out that it would be effective. Thus, in the case of glioblastoma, P144 has shown potential use by reducing proliferation, migration, invasiveness, and tumorigenicity [47]. On the other hand, it has also been seen that TGF-β is a mediator in the formation of metastases from the colon to the liver [48]. Furthermore, it has also been reported that the TGF-β is abundant in the environment of osteosarcomas and that inhibiting its production osteosarcoma progression is reduced [49]. Therefore, these collective findings support the idea that P144 could be effective in the treatment of sarcomas.

The proof of concept of a systemic formulation of Disitertide^©^, for the prevention of brachytherapy induced fibrosis, is validated in this work as a relevant strategy for future clinical applications that include other tissue locations tumors in relation with radiotherapy induced fibrosis. Furthermore, Disitertide^©^ may have potential in radiotherapy-associated fibrosis in other organs and tissues, but this hypothesis should be confirmed in further studies with suitable animal models and different preclinical proof-of-concept studies. Further studies are necessary to elucidate whether the application of Disitertide^©^ in the late phase of the radiotherapy induced fibrosis formation might avoid the excessive deposition of extracellular matrix components, hence acting as a preventive treatment.

## 5. Conclusions

In this work, we demonstrate that P144 treatment reduces RIF intensity and fibrotic tissue response in a rabbit model of brachytherapy, and this reduction is related to a decrease in the levels of Smad2/3 phosphorylation, as a representation of the canonical intracellular pathway activation of cells in response to TGF-β biological activity. These results invite the clinical consideration of a new potential co-treatment approach to reducing complications in soft tissue sarcomas treated with radiotherapy.

## Data Availability

The data presented in this study are available on request from the corresponding author.
